# Editorial

**Published:** 1898-04

**Authors:** 


### EDITORIAL DEPARTMENT.

F. E. DANIEL, M. D., Editor.
S. E. HUDSON, M. D., Managing Editor.
A. J. SMITH. M. D., Galveston, Associate Editor.

Published Monthly at Austin, Texas, by Drs. Daniel and Hudson. Subscription price $2.00 a year in advance.

Eastern Representatives: Messrs. Monihan & Fairchild, St. Paul Building, 220 Broadway, New York City.

Official organ of the West Texas Medical Association, the Houston District Medical Association, the Austin District Medical Society, the Brazos Valley Medical Association, the Galveston County Medical Society, and several others.

### EDITORIAL.

THE ADVERTISING PHYSICIAN AND THE CODE.—A physician may advertise with perfect propriety, provided he confines the wording of the advertisement within the limits prescribed by the code of ethics. It is perfectly legitimate for him to put his professional card in the newspapers; this card announcing his name, degree, and address; also his office hours and telephone connections, if he should desire to add these. He may even announce in his card that he is doing special work, or is giving his time and attention to one or more special lines of practice, provided his advertisement reads, "practice limited to," these special lines. He cannot say "special attention given to" any particular class of cases without violating the code. In other words, when he advertises in the newspapers or states on his professional card that he is doing any kind of special work, he must, in order to comply with the code, limit his practice entirely to the line or lines so advertised. He cannot do a general practice and at the same time advertise that he gives special attention to any one or more classes of patients or diseases. The custom, so prevalent with many of the younger members of the profession, of advertising "special attention given to diseases of women," or "special attention given to the treatment of chronic diseases," and all similar expressions, are violations of the code of ethics. Many of our professional brethren fall into this practice unwittingly, not knowing that it is contrary to medical ethics, and that some unscrupulous members of the profession make use of this form of advertising simply to secure all of that class of cases in addition to their general practice, thus doing a manifest injustice to their medical neighbors who have had just as good opportunities and have given as much "special attention" to these branches as the advertisers have.

The purpose of the code of ethics is the establishment of a set of rules for the guidance of medical men in their conduct to ward each other and toward their patrons. In other words, it is in reality the application of the golden rule in our professional life.

Other Objectionable Ways of Advertising.—We regret the fact that there are many physicians who are members of the regular profession, perhaps holding membership in one or more medical societies, and should, therefore, be examples of ethical conduct, who resort to means for procuring patronage which are much more objectionable that those just mentioned, and are flagrant violations of the spirit of the code. It is a curious and a deplorable fact that in many of our larger towns and cities we have one doctor who by illegitimate and unethical means manages to secure the largest part of all emergency calls or calls to attend the injured in cases of accident. These calls are usually made by members of the police force, and whether this physician divides the fee with them or gives them free medical service, or just what the consideration may be, is an open question; at any rate, there is unquestionably an understanding and a bonus. This physician who makes sure that the newspaper reporter is informed of these accident cases—in fact, of all his cases of an unusual nature—and the reporter never omits to mention that "Dr. Blank was hastily summoned and rendered prompt and skillful service, but for which the patient would have succumbed." The reporter is no doubt paid at so much for each report. This form of advertising is worse, if possible, than the untruthful newspaper boasting of the blatant quack and mountebank. The one, while willfully making use of unprofessional and dishonest methods to secure business, laboring under the delusion that he is sharper than his professional brethren and that they are too stupid to be aware of his disreputable methods, makes a pretense of practicing a noble profession in a legitimate way, and expects his fellow physicians to extend to him all the courtesies prescribed by the code; the other plies his nefarious business open and above board, expecting nothing at hands of educated physicians but contempt and condemnation. Neither of them is worthy of the respect of decent men, and both should be denounced as unscrupulous scoundrels.

Of other illegitimate ways of advertising we shall say but little at this time, leaving the subject open for discussion in some future issue of the JOURNAL. It is perhaps well enough to mention, in order to condemn, the oral forms of advertising. These are of different kinds, one being the boasting by the recent graduate of the superior hospital advantages which he has enjoyed, another by the old practitioner, of his long experience, etc., knowledge coming, he says, almost entirely from *experience.* One boasts of the number of hours of sleep he has lost on account of the great rush of professional work. Another delights in repeatedly telling of the number of calls he has made or the number of prescriptions he has written in a single day, these numbers sometimes running away up into the impossible. These things are all done for advertising purposes, and no thoroughly ethical physician and gentleman should ever make use of them.

